# An automated determination of beta-glucuronidase activity in human serum with the Abbot VP bichromatic analyzer

**DOI:** 10.1155/S1463924685000323

**Published:** 1985

**Authors:** Constantino G. Bao, Francisco V. Alvarez

**Affiliations:** 1 Servicio de Análisis Clínicos Hospital ‘San Agustin’ Avilés Asturias Spain; 2 Departmento de Bioquímica Facultad de Medicina Universidad de Oviedo Oviedo Spain


Journal of Automatic Chemistry/*Journal of Clinical Laboratory Automation*, Vol. 7, No. 3 (Jul–Sep 1985), pp. 152–154

### An automated determination of beta-glucuronidase activity in human serum with the Abbot VP bichromatic analyzer

**Constantino G. Bao and Francisco V. Alvarez**
*Servicio de Análisis Clínicos, Hospital 'San Agustin' Avilés, Asturias, Spain, and Departmento de Bioquímica, Facultad de Medicina, Universidad de Oviedo, Oviedo, Spain*

### Introduction

The determination of serum beta-glucuronidase (b-D-glucuronide glucuronohydrolase, EC 3.2.1.31) is of clinical interest because it can be used in the diagnosis of several pathological conditions. Serum levels of beta-glucuronidase are increased in patients with such conditions as neopalms [1], diabetes mellitus [2 and 3], in pregnant women and in gestational diabetes [4], atherosclerotic disease [5], coronary artery disease [6], and Gaucher's disease [7]. However, it is practically absent in sera of patients with mucopolisaccharidosis [8].

When a glycoside of a b-D-glucosiduronic acid is used as substrate, beta-glucuronidase catalyses the transfer of b-D-glucosiduronic acid to an appropriate acceptor such as water (hydrolysis reaction), alcohols and glycols.

Fluorimetric and colorimetric measurement, based on the hydrolysis reaction, can be carried out for the determination of beta-glucuronidase activity [9]. In the colorimetric methods, three substrates are widely used—the glucoronides of phenolphthalein [10], 4-nitrophenol [11], and 8-hidroxiquinoline [12], which upon enzymatic action liberate the corresponding aglycone. These are coloured substances in alkaline mediums. 4-nitrophenol-b-D-glucuronide was chosen as the substrate for the authors' beta-glucuronidase assay because 4-nitrophenol is the final product in at least 20 different enzymatic determinations [13].

In this paper, an optimized method [14] for determining the enzymatic activity of beta-glucuronidase has been adapted for the Abbott VP Bichromatic Analyzer. In the reference method, the incubation time was 1 h, the substrate concentration was 8mM in the assay and the CVs calculated were: within-run CV = 8.86 $\bar{x}$ = 1.05; CV = 4.99, $\bar{x}$ = 4.47; between-day CV = 7.75, $\bar{x}$ = 2.85.

### Materials and methods

### *Reagents*

Acetic acid, sodium acetate, and sodium hydroxide, all analytical grade reagents from Merck, Darmstadt, FR Germany. 4-nitrophenyl-b-D-glucuronide, 4-nitrophenol, b-glucuronidase from bovine liver and glycine from Sigma Chemical Co., St. Louis, Missouri, USA.

### *Apparatus*

UV-Vis spectrophotometer, Minikem with printer (Coulter Electronics, Inc., Hialech, Florida, USA). pH meter 28 (Radiometer Copenhagen, Denmark). Analytical balance H 10 W and precision balance P 1210 from Mettler Instrumente AG, Switzerland. Abbott VP Biochromatic Analyzer (Abbott Laboratories, Dallas, Texas, USA).

### *Samples*

The blood samples used were collected from healthy volunteers, blood donors, and pregnant women attending the Hospital 'San Agustin'. The sera were stored in a refrigerator at 4–8 °C until the determination of enzymatic activity was carried out. The use of heparinized plasma was avoided because heparin has been described as inhiting beta-glucuronidase activity [15]. The use of haemolysed sera was also avoided.

Two pools of sera were made, one normal and the other pathological, which were used as controls; these were stored at −20 °C. Frozen samples may be analysed if mixed throughly after thawing.

### *Method*

Before analysis the instrument is set as follows:

| | |
|---|---|
| TEST NAME | USER TEST |
| TEMPERATURE | 37 °C |
| FILTER VALUE | 415/450 |
| UNITS OF MEASUREMENT | IU/1 |
| DILUTION RATIO SETTING | 1 : 11 |
| AUXILIARY DISPENSER? | YES |
| AUXILIARY DISPENSE STATION | 11 |
| AIR MIX? | NO |
| AUXILIARY DISPENSE VOLUME | 150.34 |
| FRR? | YES |
| REACTION DIRECTION | UP |
| STANDARDS? | NO |
| REAGENT BLANK? | YES |
| ASSAY FACTOR | 696.1 |
| TEST TYPE? | END POINT |
| AUXILIARY DISPENSE REVOLUTION | 8 |
| BGN PRT REVOLUTION | 9 |
| PRT REVOLUTION | 1 |
| INITIAL REAGENT AD | 0.2 |
| UP LIMIT? | YES |
| MAX ABSORBANCE LIMIT | 1.5 |

In the test-tube, the main syringe deposits the substrate p-nitrophenyl-b-D-glucuronide (4 mM in the assay, dissolved in the buffer HAc/Ac̄ 0.1 M pH = 4.0, with 25 μl of the sample). The auxiliary dispenser is filled with buffer glycine-NaOH 0.5 M pH = 10.6. Water is placed in position No.1 of the carousel, and the different samples are placed in positions 2 through 31. In the first assays, water was deposited in the 00 position of the carousel, in order to ensure that the substrate was not undergoing hydrolysis.

The assay factor is calculated using standards containing 1.0 mM 4-nitrophenol which are processed as a normal samples. Results are thus obtained directly in IU/l.

### Experimental results

### *Analytical variables*

*Linearity*

As shown in figure 1, the method is linear over a wide range of activity values, since those up to 110 IU/l can be measured without dilution. Because these high activities are rarely found in human sera, purified beta-glucuronidase from bovine liver was used for this experiment.

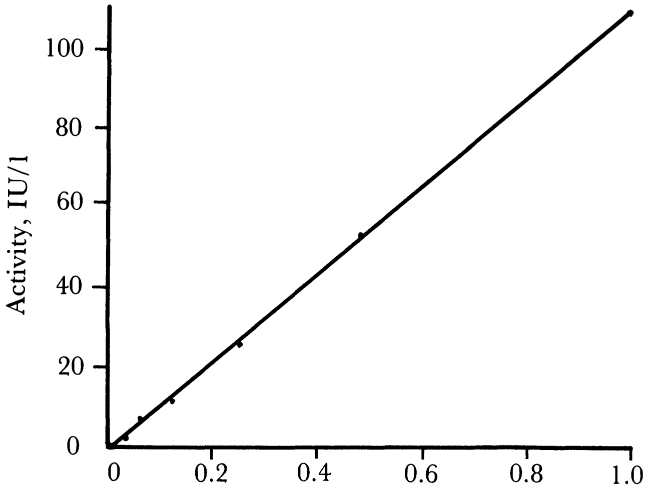

*Figure 1. Linearity of the method of assay. A sample of beta-glucuronidase from bovine liver was serially diluted with isotonic saline solution to prepare different dilutions, and the activity of the enzyme determined in all of them.*

*Precision*

Table 1 shows the precise data from the assay. Samples with normal and high activities were assayed to estimate the within-run variation. To estimate the between-day variation, the sample was divided into equal parts which were frozen at −20 °C and assayed during the following 25 days.

*Interferences*

Interferences were observed only in the haemolysed sera, as previously mentioned, and in the lipaemic sera. The interference in the latter sera is due to the cloudiness produced as a result of the low ratio of dilution (substrate/sample = 1 : 11), in which case the VP Bichromatic Analyzer gives a low energy reading.

*Table 1. Precision studies of assay beta-glucuronidase activity.*

| | Within-run | | Between-day | |
|---|---|---|---|---|
| serum value | low | high | low | high |
| $\bar{x}$, IU/l | 1.22 | 2.84 | 1.08 | 3.01 |
| SD | .057 | .035 | .061 | .105 |
| CV, % | 4.73 | 1.23 | 5.64 | 3.48 |
| N | 25 | 25 | 25 | 25 |

*Correlation with the reference method*

Statistical analysis for 40 sera samples containing beta-glucuronidase from 0.6 to 5.0 IU/l demonstrated that, in comparison with the reference method [14], the automated method performed extremely well (figure 2). The regression equation was $y = 1.047x - 0.238$, where $y$ represents the results obtained with the optimized method. The correlation coefficient was 0.978.

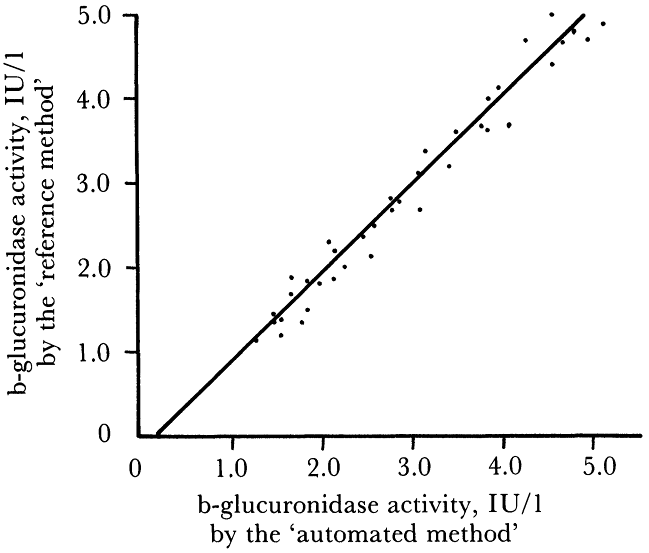

*Figure 2. Comparison of the beta-glucuronidase activity, in 40 samples, determined by the 'automated method' and by the 'reference method'.*

*Reference values*

Reference values were obtained from blood donors' sera. The total number was 267, of whom 207 were men and 60 women, with ages ranging from 18 to 59 years. Figure 3 shows the frequency distribution for men and women. The reference values for men range from 0.52 to 2.60 IU/l (average 1.56 IU/l) and for women from 0.50 to 1.80 IU/l (average 1.15 IU/l).

### Discussion

The adaptation of beta-glucuronidase analysis to the Abbott VP Bichromatic Analyzer has provided a method of analysis which is twice as fast as the reference method,

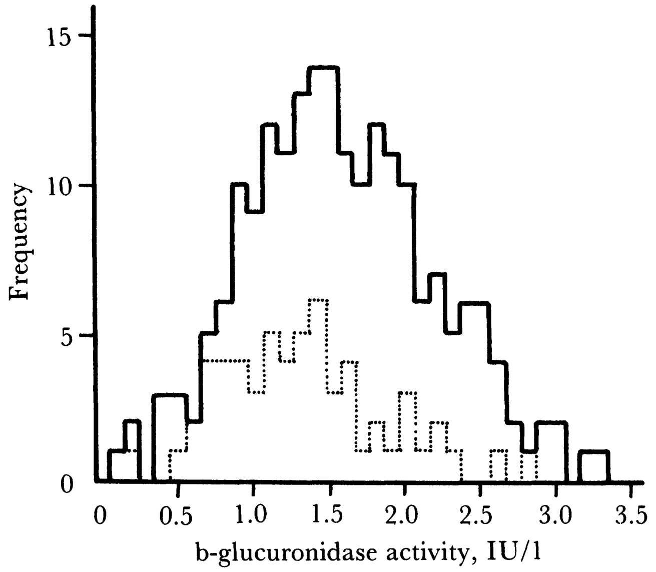

*Figure 3. Distribution of beta-glucuronidase in the sera of 60 women and 207 men in a donor's population. (. . . . .) female; (———) male.*

and more than four times faster than the rest of the methods (the time required was reduced from 2 h to 23 min). The outstanding linearity obtained with the automated method (up to 110 IU/l) has allowed a substrate concentration of 4 mM to be used in the assay, half that of the reference method, without the risk of being outside the Michaelis-Menten linear zone as it relates to the activity values of the samples used. This 50% reduction in the substrate concentration, together with a reduction in the quantities of reagents needed, make the automated method extremely economical.

The 'within-run' and 'between-day' CVs achieved with this method for two levels of activity, normal and pathological, are better than those obtained with the optimized method used as reference. Thus, by adapting the method for determining beta-glucuronidase activity to the Abbott VP bichromatic analyzer, a method was found that is more economical, more precise, and simpler to employ. The case of reproduction of the results, and the close statistical correlation with the reference method, combine to create a new and useful method of beta-glucuronidase analysis.

### Acknowledgements

We wish to thank the sales manager of Abbott in Spain, Mr Angel Medina, for his technical assistance; the laboratory technician, M. J. De los Bueis, for her invaluable help; and the rest of our technicians for their collaboration in this work.

*References*

1. Goldburg, J. A., Pineda, E. P., Banks, B. M. and Rutenberg, A. M., *Gastroenterology*, **36** (1959), 193.
2. Belfiore, F., Vecchio, L. and Napoli, E., *Clinical Chemistry*, **19** (1973), 447.
3. Miller, B. F., Keyes, E. P. and Curreri, P. W., *JAMA*, **195** (1966), 127.
4. Ferrara, M., Mizrahy, O., Saposhnisk, A. and Feinstein, G., *Israel Journal of Medical Science*, **15** (1979), 746.
5. Hagenfeldt, L. and Wahlberg, F., *Lancet* (1965), 788.
6. Miller, B. R., Keyes, E. P. and Curreri, P. W., *Journal of Atherosclerosis Research*, **7** (1967), 591.
7. Okerman, P. A. and Koklin, P., *Clinical Chemistry*, **15** (1969), 61.
8. Sly, S. W., Quinton, B. A., McAllister, H. W. and Rimoin, D. L., *Journal of Paediatrics*, **82** (1973), 249.
9. Verity, M. A., Coper, R. and Brown, W. J., *Archives of Biochemistry and Biophysics*, **106** (1964), 386.
10. Fishman, W. H., Kato, K., Anstiss, C. L. and Green, S., *Clinica Chimica Acta*, **15** (1967), 435.
11. Fishman, W. H., In *Methods of Enzymatic Analysis*, Vol. 2, Ed. H. U. Bergmeyer (Academic Press. New York, 1974), 929.
12. Wang, Ch. Ch. and Touster, O., *Journal of Biological Chemistry*, **247** (1972), 2650.
13. Bowers, G. N. Jr., McComb, R. B., Christensen, R. G. and Shaffer, R., *Clinical Chemistry*, **26** (1980), 724.
14. Bao, C. G. and Alvarez, F. V., *Diagnóstico Biológico*, **32** (1983), 29.
15. Fishman, W. H., *Advances in Enzymology*, **16** (1955), 361.

**THE AUTOMATED ANALYSIS OF IONS IN SOLUTION**

*At the University, York,*

*Wednesday 25 September to Friday 27 September 1985*

This symposium, which is being organized jointly by the Automatic Methods Group and the North East Region of the Analytical Division of the Royal Society of Chemistry, will consider the application of a wide range of automated analytical techniques (both wet chemical and chromatographic) to the analysis of ions in solution. Sessions on spectrophotometric, electroanalytical, conventional chromatographic and combination chromatographic methods have been organized and will be introduced by noted keynote speakers. An exhibition of appropriate analytical instrumentation is being arranged.

The registration fee, which will also cover light refreshments, lunch, and the evening social programme, is £60 for RSC members, £90 for non-members and £30 for retired members or students. Accommodation will be available in the University Halls of Residence, and an additional fee will be charged for overnight accommodation, dinner and breakfast.

*Further information can be obtained from Dr Clive Jackson, Health & Safety Executive, 403 Edgware Road, London NW2 6LN. Tel.: 01 450 8911.*

